# Supplementary material for: Intermolecular Coulombic decay in liquid water competes with proton transfer and non-adiabatic relaxation
Source: Nat Commun. 2025 Jul 22;16:6732. doi: 10.1038/s41467-025-61912-w (PMC12284144; doi:10.1038/s41467-025-61912-w)
Supplement: Supplementary file 1 — Supplementary Information [file 41467_2025_61912_MOESM1_ESM.pdf]

Supplementary Information for: Intermolecular Coulombic decay in liquid water competes with proton transfer and non-adiabatic relaxation

Pengju Zhang[1,2], Joel Trester[2], Jakub Dubský[3], Přemysl Kolorenč[4], Petr Slavíček[3] and Hans Jakob Wörner[2]

[1]Beijing National Laboratory for Condensed Matter Physics and Institute of Physics, Chinese Academy of Sciences, Beijing 100190, China.
[2]Laboratory of Physical Chemistry, ETH Zürich, Zurich, 8093, Switzerland.
[3]Department of Physical Chemistry, University of Chemistry and Technology, Prague, Czech Republic.
[4]Charles University, Faculty of Mathematics and Physics, Institute of Theoretical Physics, V Holešovičkách 2, 18000 Prague, Czech Republic.

Corresponding authors: pengju.zhang@iphy.ac.cn; petr.slavicek@vscht.cz; hwoerner@ethz.ch;

Supplementary Note 1: Confirmation of ICD with negative bias potential

Since ICD electrons are generally slow (0 to 10 eV kinetic energy), on the one hand, the variation of the detection efficiency of the slow electrons ($<$ 1 eV) will modulate their measured energy distribution. On the other hand, the electrons emitted from the vapor surrounding the liquid jet can lead to false coincidence events, which influences the determination of the occurrence of ICD. In principle, the false-coincidence events in the latter case should be sufficiently hindered under the experimental conditions of low count-rate, where the correlated electron pairs are essentially created through a single photoionization process. Therefore, in order to avoid possible errors coming from the unknown detection efficiency of the slow electrons ($<$ 1 eV) and to examine the capability of low count-rate measurements to suppress the contribution from gas-phase water [1, 2], we performed additional measurements with a bias potential of -20 V applied to the liquid jet. Under the negative bias potential, the electrons originating from the liquid bulk will be accelerated towards the higher kinetic energy region ($\geq$ 20 eV), which overcomes the deceasing detection efficiency for slow electrons ($<$ 1 eV), ensuring a relatively constant detection efficiency for both two liquid samples and enabling the comparison between the measured spectra. Meanwhile, the electrons liberated from the vapor will only be partially accelerated some distance away from the liquid surface. Consequently, the gas-phase signal is energetically smeared out and therefore separated from the liquid-phase electrons. The experimental results are shown in Supplementary Figure 1, where the XUV energy is chosen to be 79 eV. Within the same data acquisition time, the total coincidence counts are less than the results with a small positive bias potential (see Figure 1 in the main text). This is mainly caused by the slightly decreased collection efficiency as the kinetic energy of the electron increases[3]. Nevertheless, both the 2D correlated maps and the 1D electron spectra confirm that the observation of ICD can be accessed under the low count-rate long-term measurement, regardless of the polarity of the bias potential.

Supplementary Note 2: Determination of the ICD efficiency

In the following, we address the detailed information on the derivation of the equations that links the experimental spectra and the ICD efficiency. The ICD process is triggered by photoionization of the $2a_1$ band of liquid water, which firstly creates a photoelectron of kinetic energy E_{2a_1}. The relaxation of the excited vacancy states transfers the extra energy to a neighboring molecule and liberates a slow ICD electron with kinetic energy E_{ICD}. Under the present experimental conditions, the XUV photon energies are chosen to make sure that the kinetic energy of the $2a_1$ electron (E_{2a_1}) is much larger than that of the ICD electron (E_{ICD}), *i.e.*, $E_{2a_1} > E_{\mathrm{ICD}}$, which allows us to distinguish these

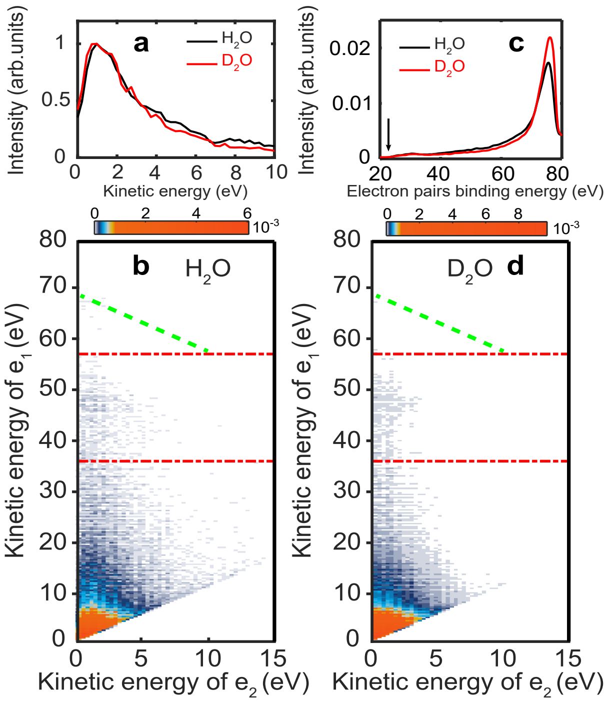

Supplementary Figure 1: Coincidence measurements of ICD in liquid H_2O and D_2O with XUV photons of 79 eV, the liquid jet was biased with a negative potential -20 V (see the experimental details in the main text), all of the energy axes were corrected by this value. Panel **a** Comparison of the energy spectra of ICD electrons from liquid H_2O (black solid) and D_2O (red solid). The energy spectra are obtained by integrating over the rectangle area ($e_1 \in [36, 57]$ eV) from panels **b** and **d**, respectively. The spectra are normalized to their maximum for comparison. Panel **c** Comparison of the electron-pair spectra of liquid H_2O (black solid) and D_2O (red solid) as a function of their electron-pair binding energy, obtained by summing along lines of constant total energy ($E(e_1)+E(e_2)$), i.e. lines parallel to the green dashed lines in panels **b** and **d**, respectively. The spectra are normalized to its total counts for comparison. Panels **b, d** Coincidence map of electron pairs produced by ionization of inner-valence $2a_1$ band in liquid H_2O and D_2O (note the logarithmic intensity scale), respectively. The area between the two red lines is dominated by $2a_1$-photoelectron/ICD-electron pairs.

two electrons experimentally. In general, the efficiency ξ of an autoionization process including two emitted electrons can be rigorously written as:

$$\xi = \frac{P(e_1, e_2)}{P(e_1)} \tag{1}$$

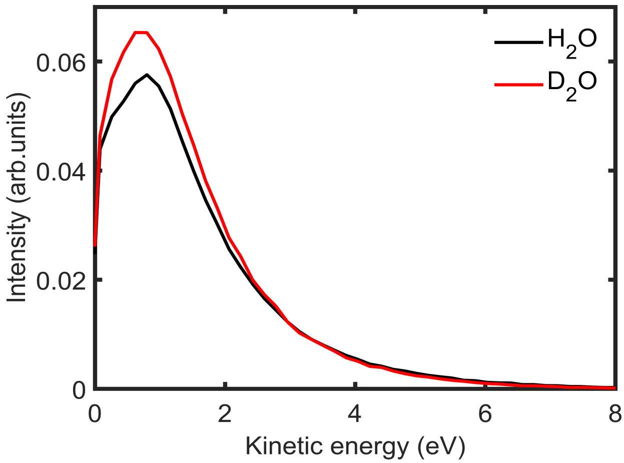

Supplementary Figure 2: Comparison of the low-energy electron-pairs distribution emitted from liquid H2O (black solid line) and D2O (red solid line), respectively. The spectra are normalised to the total electron-pair counts for comparison.

where e_1, e_2 are the fast photoelectron and slow electron, respectively. $P(\mathrm{e}_1,\mathrm{e}_2)$ is the detection probability of the electrons e_1 and e_2 simultaneously, $P(\mathrm{e}_1)$ represents the detection probability of the first electron e_1 with and without the formation of the second electron e_2. Regarding our experiment, since $E_{2a_1} > E_{\mathrm{ICD}}$, we can reasonably apply this equation to derive the ICD efficiency.

In terms of the liquid-phase measurement, the complexities drive us to consider several corrections to equation (1) as follows:

1. the detection efficiency of the photoelectron spectrometer,
2. the water vapor surrounding the liquid jet,
3. the depletion by inelastic electron scattering in the bulk and the vapor.

Considering the first point, we have to multiply an instrumental detection efficiency γ to both the ICD electrons and the photoelectrons, which results in a modified equation for the ICD efficiency:

$$\xi_{\mathrm{ICD}} = \frac{\gamma(E_{\mathrm{ICD}}) \cdot P(E_{2\mathrm{a}_1}, E_{\mathrm{ICD}})}{P(E_{2\mathrm{a}_1})} \tag{2}$$

where ξ_{ICD} represents the ICD efficiency, $\gamma(E_{\mathrm{ICD}})$ is the detection efficiency of the ICD electron. Importantly, the detection efficiency of the fast photoelectron $E_{2\mathrm{a}_1}$ appears in both the numerator and the denominator and therefore cancels out.

Considering the second point, the spectrum has to be disentangled into two contributions, the major part comes from liquid, the minor part comes from the surrounding vapor. Therefore, the detection probability of the photoelectron

$P(E_{2a_1})$ can be written as:

$$P(E_{2a_1}) = \gamma^{\text{liquid}}(E_{2a_1}) \cdot Y^{\text{liquid}}(E_{2a_1}) + \gamma^{\text{gas}}(E_{2a_1}) \cdot Y^{\text{gas}}(E_{2a_1}) \quad (3)$$

where Y^{liquid} and Y^{gas} represent the photoelectron yields of liquid and gas, respectively. γ^{liquid} and γ^{gas} denote the corresponding detection efficiencies of the photoelectrons originating from liquid and gas, respectively. Since the ionization potentials of liquid and gas are very close, we can have γ^{liquid}=γ^{gas}. Similarly, the detection probability of electron pairs $P(E_{2a_1}, E_{\text{ICD}})$ can be written as:

$$\begin{aligned} P(E_{2a_1}, E_{\text{ICD}}) = \gamma(E_{2a_1}) \cdot Y^{\text{liquid}}(E_{2a_1}) \cdot \xi_{\text{ICD}}^{\text{liquid}} \cdot Y^{\text{liquid}}(E_{\text{ICD}}) \\ + \gamma(E_{2a_1}) \cdot Y^{\text{gas}}(E_{2a_1}) \cdot \xi_{\text{ICD}}^{\text{gas}} \cdot Y^{\text{gas}}(E_{\text{ICD}}) \end{aligned} \quad (4)$$

where $\xi_{\text{ICD}}^{\text{liquid}}$ and $\xi_{\text{ICD}}^{\text{gas}}$ represent the ICD efficiency of liquid and gas, respectively. Given the experimentally confirmed negligible contribution of water clusters in the vapor surrounding the liquid jet under high-vacuum conditions [4, 5], we attribute the observed ICD process exclusively to the photoionisation of liquid water, and therefore $\xi_{\text{ICD}}^{\text{gas}}$=0.

Comparing with the gas phase, we believe that the depletion of the photoelectron via inelastic scattering is dominant in the condensed bulk liquid. We describe the inelastic losses by a factor f=1-(depletion fraction), where f^{liquid}<1, f^{gas}=1.

Combining equation (3) and equation (4), the ICD efficiency can be written as:

$$\frac{P(E_{2a_1}, e_{\text{ICD}})}{P(E_{2a_1})} = \frac{\gamma(E_{2a_1}) \cdot Y^{\text{liquid}}(E_{2a_1}) \cdot f^{\text{liquid}} \cdot \xi_{\text{ICD}} \cdot Y^{\text{liquid}}(E_{\text{ICD}})}{\gamma(E_{2a_1}) \cdot Y^{\text{liquid}}(E_{2a_1}) \cdot f^{\text{liquid}} + \gamma(E_{2a_1}) \cdot Y^{\text{gas}}(E_{\text{ICD}})} \quad (5)$$

Due to the photoelectron yield of liquid water is much larger than that of gas vapor, *i.e.*, $\frac{Y^{\text{gas}}(e_{2a_1})}{Y^{\text{liquid}}(e_{2a_1})} \ll 1$, we finally have the expression of ICD efficiency:

$$\xi_{\text{ICD}} = \frac{P(E_{2a_1}, E_{\text{ICD}})}{P(E_{2a_1})} \cdot \frac{1}{\gamma(E_{\text{ICD}})} \quad (6)$$

In order to obtain the absolute ICD efficiency, one needs to know the absolute instrumental detection efficiency of the ICD electrons. As we mentioned above, the ICD electron spectrum varies from zero to 10 eV, where the corresponding transmission efficiency of the magnetic bottle time-of-flight spectrometer is less than 100% [6] and also sensitive to the specific configuration of the magnetic fields induced by the permanent magnet together with the Helmholtz coils in the flight tube. Therefore, a reliable efficiency can only be achieved by a well-calibrated spectrometer, in particular for the slow electrons. Moreover, the spectrometer has to maintain an identical configuration

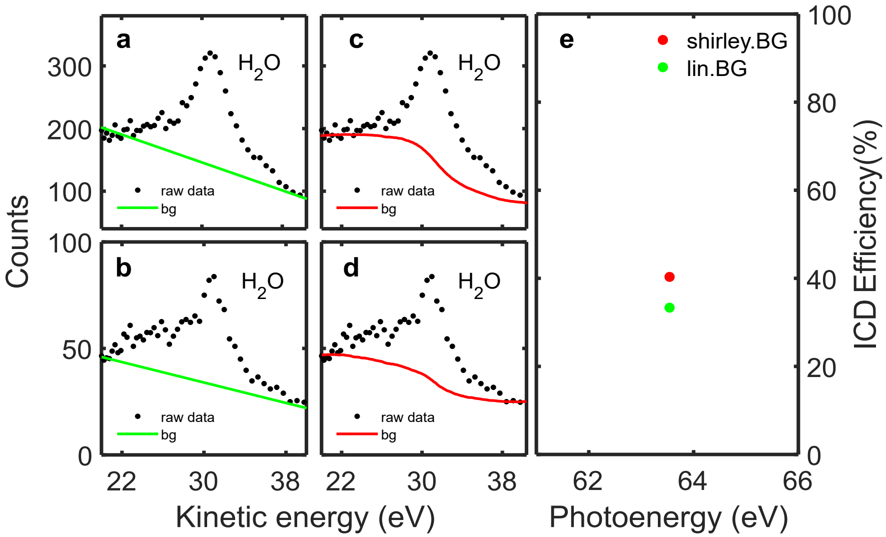

Supplementary Figure 3: Illustration of the background subtraction in the experimental data used to determine the relative efficiency of ICD. Coincident (lower panels) and non-coincident (upper panels) electron spectra recorded after photoionization of the $2a_1$ band in liquid water at hν= 63.5 eV. Panel **a** displays the intensity of photoelectrons ($P(E_{2a_1})$) recorded in the region of the $2a_1$ binding energy. Panel **b** shows $P(E_{2a_1}, E_{\mathrm{ICD}})$, the integral of the double-hit spectrum over the entire energy range of the ICD electrons ([0 10] eV), namely, the intensity of photoelectrons recorded in coincidence with an ICD electron. The green solid lines in panels **a** and **b** indicate a linear background subtraction, whereas panels **c** and **d** show a curved, Shirley-type background subtraction in red. The statistic uncertainty originates from the integration of $2a_1$ binding energies, which influences H_2O and D_2O equally. Panel **e** shows the absolute ICD efficiencies obtained by linear background subtraction (green dot) and Shirley-type background subtraction (red dot), assuming the detection efficiency of ICD electrons is unity.

for the long-term measurement. These complexities essentially hinder the extraction of the absolute ICD efficiency of liquid water. They do, however, not affect the determination of the relative ICD efficiencies.

Finally, values of the coincident spectrum intensity $P(E_{2a_1}, E_{\mathrm{ICD}})$ and non-coincident spectrum intensity $P(E_{2a_1})$ in equation (6) were determined from the number of events with electrons recorded in some energy intervals. The raw coincident and non-coincident spectra are shown in Supplementary Figure. 3, together with the illustration of the background subtraction. After the background subtraction, we obtain $\xi_{\mathrm{ICD}}^{\mathrm{H_2O}}$ and $\xi_{\mathrm{ICD}}^{\mathrm{D_2O}}$, and therefore the relative efficiency $\gamma_{\mathrm{ICD}}(\mathrm{H_2O}/\mathrm{D_2O})$, which is shown in Figure 2 (e) in the main text.

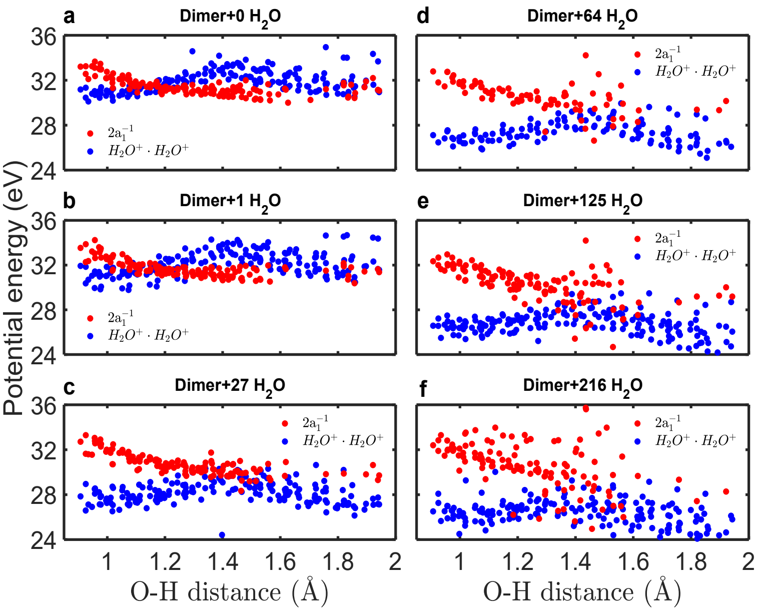

Supplementary Figure 4: Calculated energies of the singly ionized $2a_1$ state (in red) and the doubly ionized cationic states (in blue) are presented, where both O-H bonds in the first water molecule elongate. The bond that is not involved in proton transfer elongates at half the rate compared to the primary bond. The x-axis represents the distance of the transferred proton from the oxygen atom of the first water molecule. **a** Calculated energies of the singly ionized $2a_1$ (red) and doubly ionized cationic states (blue) for water dimer. **b, c, d, e, f** Calculated energies for water dimer associated with 1, 27, 64, 125 and 216 water molecules, respectively. The oscillating structure in the data are an artifact resulting from the variations in the initial OH bond distances.

Supplementary Note 3: Energetics of solvated water

The potential energy curves presented in the main text consider only proton transfer motion, keeping the positions of all other atoms fixed. However, dynamical simulations of the water dimer ionized to the $(2a_1)^{-1}$ state reveal that even the second O–H bond in the ionized unit undergoes elongation. Supplementary Figure 4 illustrates the energy dependence for both singly and doubly ionized species when allowing simultaneous hydrogen motion along the proton transfer coordinate and dissociation of the second hydrogen atom. These potential energy curves resemble those in the main text but exhibit a slightly steeper slope.

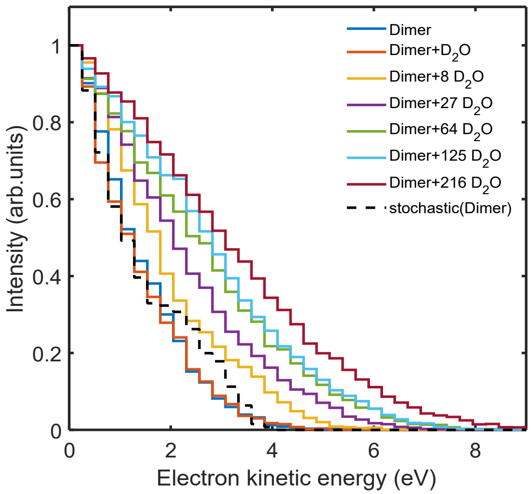

Supplementary Figure 5: Comparison of the Monte-Carlo approach using the actually simulated trajectories with a model sampling the distance distributions from fitted functions. The explicit method utilizes trajectories from molecular dynamics simulations, allowing direct evaluation of energetics from these trajectories since only a single dimer is involved. The spectra are normalized to their maximum.

Supplementary Note 4: Benchmarking the Monte-Carlo technique

For a water dimer, we can explicitly calculate the position distribution of the atoms, calculating the energetics for both molecular ions each time. This calculation provides a benchmark for the multi-scale model, using the distribution of O–H distances in the dimer. We observe a reasonable agreement between these two models, enabling us to estimate the error in the Monte-Carlo simulations. The comparison is shown in Supplementary Figure 5.

Supplementary Note 5: Fitting the model parameters

The Monte Carlo model contains two parameters that are difficult to access: the ICD lifetime and relaxation rate. Supplementary Figure 6 shows a comparison of the calculated ICD efficiencies with cluster experiments for different combinations of these parameters.

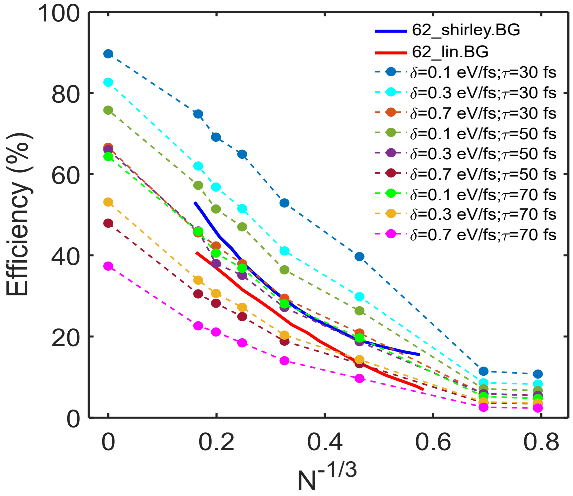

Supplementary Figure 6: Comparison of different pairs of δ and τ with experiment from Ref. [7].

Supplementary References

[1] Thürmer, S., Malerz, S., Trinter, F., Hergenhahn, U., Lee, C., Neumark, D.M., Meijer, G., Winter, B., Wilkinson, I.: Accurate vertical ionization energy and work function determinations of liquid water and aqueous solutions. Chem. Sci. **12**(31), 10558–10582 (2021).

[2] Gadeyne, T., Zhang, P., Schild, A., Wörner, H.J.: Low-energy electron distributions from the photoionization of liquid water: a sensitive test of electron mean free paths. Chem. Sci. **13**(6), 1675–1692 (2022).

[3] Kothe, A., Metje, J., Wilke, M., Moguilevski, A., Engel, N., Al-Obaidi, R., Richter, C., Golnak, R., Kiyan, I.Y., Aziz, E.F.: Time-of-flight electron spectrometer for a broad range of kinetic energies. Rev. Sci. Instrum. **84**(2), 023106 (2013).

[4] Hartweg, S., Yoder, B.L., Garcia, G.A., Nahon, L., Signorell, R.: Size-resolved photoelectron anisotropy of gas phase water clusters and predictions for liquid water. Physical Review Letters **118**(10), 103402 (2017).

[5] Faubel, M.: Early microjet experimentation with liquid water in vacuum. Accounts of Chemical Research **56**(6), 625–630 (2023).

[6] Mucke, M., Förstel, M., Lischke, T., Arion, T., Bradshaw, A.M., Hergenhahn, U.: Performance of a short "magnetic bottle" electron spectrometer. Rev. Sci. Instrum. **83**(6), 063106 (2012).

[7] Richter, C., Hollas, D., Saak, C.-M., Förstel, M., Miteva, T., Mucke, M., Björneholm, O., Sisourat, N., Slavíček, P., Hergenhahn, U.: Competition between proton transfer and intermolecular coulombic decay in water. Nat. Commun. **9**(1), 4988 (2018).
